# *MLH1* Methylation Status and Microsatellite Instability in Patients with Colorectal Cancer

**DOI:** 10.3390/genes16020182

**Published:** 2025-02-02

**Authors:** Manuel Alejandro Rico-Méndez, Miguel Angel Trujillo-Rojas, María de la Luz Ayala-Madrigal, Jesús Arturo Hernández-Sandoval, Anahí González-Mercado, Melva Gutiérrez-Angulo, José Geovanni Romero-Quintana, Jesús Alonso Valenzuela-Pérez, Ruth Ramírez-Ramírez, Beatriz Armida Flores-López, José Miguel Moreno-Ortiz

**Affiliations:** 1 Doctorado en Genética Humana e Instituto de Genética Humana “Dr. Enrique Corona Rivera”, Departamento de Biología Molecular y Genómica, Centro Universitario de Ciencias de la Salud, Universidad de Guadalajara, Guadalajara C.P. 44340, Jalisco, Mexico; manuel.rico8557@alumnos.udg.mx (M.A.R.-M.); miguel.trojas@alumnos.udg.mx (M.A.T.-R.); luz.ayala@academicos.udg.mx (M.d.l.L.A.-M.); qfb_arturohernandez@hotmail.com (J.A.H.-S.); anahi.gonzalez@academicos.udg.mx (A.G.-M.); 2 Departamento de Ciencias de la Salud, Centro Universitario de los Altos, Universidad de Guadalajara, Tepatitlán de Morelos C.P. 47600, Jalisco, Mexico; melva.gutierrez@academicos.udg.mx; 3 Facultad de Ciencias Químico Biológicas, Universidad Autónoma de Sinaloa, Ciudad Universitaria, Culiacán C.P. 80010, Sinaloa, Mexico; geovanniromero@uas.edu.mx; 4 Hospital Civil de Guadalajara “Dr. Juan I. Menchaca”, Guadalajara C.P. 44340, Jalisco, Mexico; dr_jvalenzuela@hotmail.com; 5 Departamento de Biología Molecular, Centro Universitario de Ciencias Biológicas y Agropecuarias, Universidad de Guadalajara, Zapopan C.P. 45200, Jalisco, Mexico; ruth.ramirez@academicos.udg.mx; 6 Departamento de Ciclo de Vida, Facultad de Medicina, Universidad Autónoma de Guadalajara, Zapopan C.P. 45129, Jalisco, Mexico

**Keywords:** methylation, *MLH1*, microsatellite instability, colorectal cancer

## Abstract

**Background/Objectives:** The purpose of the current study was to compare the methylation of five regions of the CpG island of *MLH1* with the presence of microsatellite instability (MSI) in colorectal cancer (CRC) patients. **Methods:** The study analyzed 138 CRC tumor samples. DNA extraction was performed, followed by bisulfite conversion. *MLH1* gene methylation was assessed by methylation-specific PCR (MS-PCR), and the resulting fragments were analyzed using polyacrylamide gels. MSI was evaluated using multiplex PCR, and the fragments were run through capillary electrophoresis. R studio (v4.4.1) and SPSS (v29.0) software were used for the statistical analysis, and values of *p* < 0.05 were considered statistically significant. **Results:** The study showed 75.4% unmethylated, 21% partially methylated, and 3.6% fully methylated samples, with region A frequently methylated. MSI was observed in 7.2% of cases (MSI-H: 5.8%, MSI-L: 1.4%). BAT-26 was the most unstable marker. A significant difference between *MLH1* methylation and MSI-H (*p* < 0.01) was identified, but there was no relationship with specific *MLH1* regions. **Conclusions:** No differences were identified when analyzing specific methylation regions in relation to MSI. This study is the first to describe MSI frequency in Mexican patients regardless of age.

## 1. Introduction

Colorectal cancer (CRC) is the third most common type of tumor and the second leading cause of cancer-related mortality worldwide [1]. This disease is caused by the interaction of genetic and epigenetic alterations, most of them related to the WNT, EGFR, and DNA repair molecular pathways, including the mismatch repair system (MMR), with the MLH1 protein being an important part of this system [2]. The mutL protein homolog 1 (MLH1) (uniprot P40692) is encoded by the gene of the same name (NIH Gene ID: 4292) [*Homo sapiens* (human)], located at 3p22.2. It is regulated by a promoter region of 1781 bp [3], which comprises a 1,128 bp CpG island containing 93 CpG sites [4]. MLH1 has a fundamental role in the MMR, and it is responsible for correcting single-nucleotide variants and insertions/deletions (indels) made by DNA polymerases during DNA replication. This mechanism contributes to maintaining the stability of the human genome [5].

Epigenetic inactivation of *MLH1* by methylation of CpG sites in the promoter region suppresses gene expression, leading to loss of protein expression, which results in a deficiency in the MMR system (dMMR), promoting CRC development and microsatellite instability (MSI) [6]. Microsatellites are short sequences that range from one to six base pairs, consisting of tandem repeats. Due to this characteristic, DNA polymerases tend to make slippage errors during replication, causing indels in their sequences. The MMR system is responsible for correcting these errors. However, the loss of function of this system leads to the accumulation of indels, altering the length of the microsatellites, a phenomenon known as MSI [7]. Microsatellites serve as markers for many sporadic and hereditary cancers [8]. In sporadic CRC, it is estimated that *MLH1* methylation and MSI occur in 19% [9] and 12% of cases [10], respectively, and the main factor responsible for the presence of MSI in sporadic CRC is the *MLH1* promoter methylation [11].

Due to the *MLH1* CpG island being too large, most studies only consider the analysis of a portion of the island. Previous studies proposed a subdivision of the CpG island from four to five regions [12,13,14,15,16] to cover most CG dinucleotides, and only Deng et al. (1999, 2001) [15,16] found a relationship between the methylation status of four regions of the CpG island and the inhibition of MLH1 expression in CRC cell lines. It has traditionally been suggested that the “C” region of the *MLH1* promoter (located −248 to −178) is the most relevant for the transcriptional activation of the gene since its methylation correlates with a lack of MLH1 expression [17]. However, other authors have suggested that full promoter methylation is required to generate MSI [18]. Therefore, the purpose of the current study is to compare the methylation of five regions of the CpG island of *MLH1* with the presence of MSI in CRC patients.

## 2. Materials and Methods

### 2.1. Study Population

A total of 138 fresh tumor tissue samples were obtained from patients with histopathological diagnoses of CRC at the Hospital Civil de Guadalajara “Dr. Juan I. Menchaca”. All patients provided written informed consent prior to their inclusion in the study, ensuring adherence to ethical standards. The research protocol was reviewed and approved by the Local Bioethics Committee (CI-01417), ensuring compliance with national and international ethical guidelines for research involving human subjects. The patients were Mexican, with at least two generations of Mexican ancestry, and were over eighteen years old. Clinical and sociodemographic information about the patients was obtained through a review of their medical records. Furthermore, all participants underwent an interview and completed a questionnaire, which facilitated the data collection on medical history and lifestyle factors.

### 2.2. DNA Extraction

DNA extraction from 25–50 mg of fresh tumor tissue was carried out using the High Pure PCR Template Preparation kit (product no.: 11796828001, Roche Diagnostic GmbH, Mannheim, Germany). The DNA was quantified by spectrophotometry and stored at −20 °C until its use.

### 2.3. DNA Bisulfite Conversion

Bisulfite conversion was made using 5 μL of DNA (100 ng/μL) treated with the *EZ* DNA Methylation-Gold^TM^ kit (product no.: D5006; ZYMO Research, Irving, CA, USA) according to the manufacturer’s instructions. Additionally, human methylated and unmethylated DNA controls from the HCT116 DKO cell line were used during DNA conversion to assess the reaction efficiency (product no.: D5014; ZYMO Research).

### 2.4. Methylation-Specific PCR (MS-PCR)

**Figure 1:**
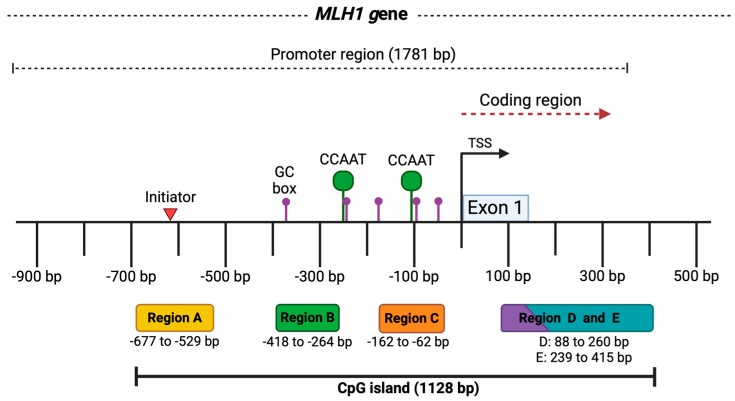
*MLH1* gene promoter region. The image represents the promoter of the *MLH1* gene with a length of 1781 bp and some key elements. At the bottom, the five analyzed regions of the CpG island are shown in colored boxes, along with the fragment size in base pairs for each one. At the top, above the black line, the locations of regulatory elements in the *MLH1* promoter are outlined, such as the initiator indicated by the red triangle (−620 bp), the GC and CCAAT boxes represented by the purple (−385, −244, −174, −90, −58 bp) and green dots (−250, −113 bp), respectively. The transcription start site (TSS) is marked by the black arrow, and exon 1 in the blue box. Taken from the *Eukaryotic Promoter Database* [19]. Created by BioRender.

The MS-PCR reactions for all assays were performed using 100 ng/µL of DNA converted with sodium bisulfite in a volume of 12 µL mixed with 1X PCR buffer (500 mM KCl, 100 mM Tris-HCl, and 0.1% Triton™ X-100), 1.5 mM MgCl₂, 2 mM dNTPs, 10 pmol of each primer, and 0.25 U/μL of Platinum Taq DNA polymerase. Previously treated controls for methylated and unmethylated DNA were included in PCR reactions. Primers for methylated and unmethylated DNA for the five regions in the *MLH1* CpG island and PCR conditions can be found in Supplementary S1 (Figure 1) [12]. Initial denaturation was carried out at 95 °C for 5 min, followed by 94 °C for 45 s, alignment at 57 °C for 45 s, and elongation at 72 °C for 1 min. This was carried out for 30 cycles.

### 2.5. Interpretation of Methylation Status

A methylated sample (M) was considered when the five regions of the CpG island were methylated, partially methylated (PM) when one to four regions were methylated, and when no region was methylated, it was considered as an unmethylated sample (UM). The MS-PCR products were visualized using 6% polyacrylamide gels stained with silver nitrate (AgNO_3_).

### 2.6. Microsatellite Instability Analysis

MSI analysis (MSA) was performed by multiplex PCR using the *Type-it Microsatellite PCR* kit (Qiagen ID. 206243) with a panel of five markers: NR-27, NR-21, NR-24, BAT-25, and BAT-26. The sequence of the primers and additional information can be found in Supplementary S1. PCR reactions were performed using 100 ng/µL of DNA in a volume of 12 µL. A primer mix was used with a concentration of 100 µM from each primer. The PCR conditions included an initial denaturation at 95 °C for 5 min, then 95 °C for 30 s, 58 °C for 90 s, and 72 °C for 30 s for 40 cycles, and a final extension at 60 °C for 30 min. Then, 2 μL of PCR products were taken and mixed with formamide and GeneScan 500 LIZ Dye Size Standard (Thermo Fisher, Waltham, MA, USA). Samples were heated at 96 °C for 5 min followed by a thermal shock on ice, and after that, the samples were analyzed by the SeqStudio Genetic Analyzer (Applied Biosystems, Carlsbad, CA, USA). MSA interpretation was made using the Microsatellite Analysis Thermo Fisher Connect^TM^ software v1.0. Two or more unstable markers were defined as high MSI (MSI-H), an unstable marker was determined as low MSI (MSI-L), and no unstable marker as microsatellite stability (MSS).

### 2.7. Statistical Analysis

Quantitative variables were presented as means and standard deviations, while qualitative variables were presented as percentages. Proportions between groups were compared using the chi-square test/Fisher’s exact test. For multiple comparisons, Bonferroni correction was applied when a significant *p*-value was observed. Values of *p* < 0.05 were considered significant. Statistical analyses were performed with R studio (v4.4.1) and SPSS (v29.0).

## 3. Results

Tumor tissue samples from 138 Mexican patients with CRC were analyzed and were predominantly male. Clinical and pathological characteristics of these patients are presented in Table 1.

**Figure 2:**
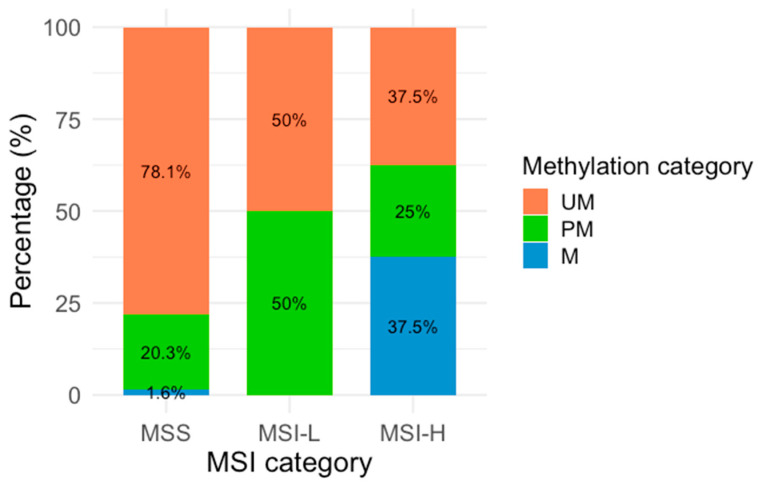
*MLH1* methylation across MSI categories. Percentages represent the distribution of each methylation category within the MSI categories. The *X*-axis represents the three MSA categories (MSI-H: Microsatellite Instability High, MSI-L: Microsatellite Instability Low, and MSS: Microsatellite Stability), and the *Y*-axis is represented by colors corresponding to the three methylation categories: orange (UM: Unmethylated), green (PM: Partially methylated), and blue (M: methylated).

Regarding the methylation analysis, it was found that 3.6% (n = 5) of the samples were methylated, 21% (n = 29) partially methylated, and 75.4% (n = 104) unmethylated. In the MSA, 92.8% (n = 128) of the samples were classified as MSS, while 7.2% (n = 10) showed MSI, comprising 5.8% (n = 8) MSI-H and 1.4% (n = 2) MSI-L. The analysis of *MLH1* methylation status was stratified according to the MSI classification (Table 2 and Figure 2). Figure 2 shows the proportional distribution of M, PM, and UM samples according to the MSI category. Among methylated samples, three were classified as MSI-H, and BAT-26 was identified as the most frequently unstable marker.

Regarding the analysis of methylation regions (Supplementary S2), findings revealed notable variability among the eight MSI-H samples. Specifically, three of these samples exhibited methylation across all analyzed regions. In contrast, only two samples showed methylation exclusively in region A.

We also performed a comparison between *MLH1* methylation and MSA results with the clinicopathological characteristics shown in Table 1; nonetheless, no significant differences were identified. The only significant differences observed were in the comparison of MSI status between two age groups (<<50 and >50 years) (*p* < 0.001).

## 4. Discussion

CRC is characterized by a genetic instability where tumor suppressor gene inactivation plays a critical role in tumor development [2]. *MLH1* gene is considered a tumor suppressor gene as it contributes to the maintaining of genetic stability; however, in CRC, MLH1 expression is commonly lost, either due to genetic or epigenetic alterations [6]. In this study, we evaluate the methylation status of *MLH1* by comparing CpG methylation in five regions of *MLH1* with the presence of MSI in CRC patients.

This study examined 138 tumor samples from Mexican patients with CRC. As the literature reports, most patients were male (56.5%) in contrast to female patients, and the mean age was 57 years. Our results showed that 75.4% of samples were unmethylated, 21% were partially methylated, and 3.6% methylated. Although the frequency of DNA methylation observed in the present study appears to be the lowest, it is also the most comprehensive in terms of the extent of the analyzed CpG island in *MLH1*, as most authors typically examine only a single region. Several authors have reported a variety of proportions ranging from 5.2% to 50% of methylated samples in different populations [13,20,21,22,23,24,25,26]. In the Mexican population, we previously reported a methylation frequency of 24.8%; however, a subdivision of methylated and partially methylated was not considered [12].

In the MSA, our study reports an MSI frequency of 7.2% (MSI-H: 5.8%, MSI-L: 1.4%) and 92.8% of MSS. Evidence shows frequencies from 5.5% to 43.3% of MSI in sporadic CRC in several populations around the world [27,28,29,30]. However, those studies also differ in the number of patients analyzed and markers included. An MSI frequency of 21.3% (MSI-H: 14.9%, MSI-L: 6.4%) was reported in 47 Mexican patients with CRC [31]. A comparison of the proportions between both populations was conducted, revealing a significant difference (*p* = 0.0073). Despite analyzing almost the same set of markers (excluding the NR-27 marker) and applying the same interpretation for MSI classification, the observed differences may be explained by the fact that all patients included in that study were under 50 years old, suggesting a potential hereditary component since most hereditary CRCs are due to Lynch syndrome, of which 95% of cases are MSI-positive [32]. This work is the first study to analyze MSI in Mexican populations despite the age of patients.

On the other hand, it is notable that the BAT-26 marker was the most frequently unstable in MSI-H samples, which aligns with its high sensitivity and specificity reported in previous studies as an indicator of MSI in CRC [33,34]. Additionally, it has the capability to detect MSI in samples with a tumor cell content as low as 5–10% [35]. Similarly, it is important to emphasize that the MSI panel employed in our study is highly reliable, as it was validated for CRC, showing a sensitivity of 95.6% and achieving 100% specificity and predictive value [34]. This is relevant because it is well-established and FDA-approved that patients with CRC and other unresectable or metastatic tumors with MSI-H positive values respond favorably to monoclonal antibody (mAB) therapies, such as pembrolizumab, targeting immune checkpoints like programmed cell death protein 1 (PD-1) or programmed cell death ligand 1 (PD-L1) [36]. Since patients with MSI-H express many tumor-specific antigens due to the high mutation burden caused by dMMR, this triggers an immune response mediated by cytotoxic T cells, whose effector phase is downregulated by the recognition of PD-1 on T cells and PD-L1 on tumor cells. Therefore, the inhibition of this interaction by mAB reactivates the effector phase, allowing T cells to attack MSI-H positive tumor cells [7]. This underlines the need to perform MSA in CRC patients as it could have an impact on better management and prognosis.

Although we performed statistical analysis to evaluate the relationship between MSI and clinicopathological characteristics, we did not find any significant result; nevertheless, MSI usually relates to right and poorly differentiated tumors [9].

Overall, our findings revealed significant differences between *MLH1* methylation status and MSI (*p* = 0.01), aligning with previous studies [14,37,38,39,40] that highlight the critical role of MLH1 and the impact of its DNA methylation on genetic instability. Nonetheless, further research is needed to evaluate MLH1 protein expression to elucidate the specific influence of methylation in individual regions and its effect on expression levels and MSI development. Our results indicated that there was no significant difference between the methylation of any specific region and MSI, and no other study has linked these variables. In terms of protein expression, Deng et al. (1999, 2001) [15,16] analyzed the methylation of four regions of the *MLH1* promoter (A, B, D, E). However, the regions defined by the primers used in these studies are not the same as this study, and additionally, their analyses were based on cell lines. They reported that methylation in the regions they designated as A (−711 to −577) and B (−552 to −266) is not associated with the loss of MLH1 protein expression. This finding could be related to our results (see Supplementary Material), where the majority of MSS samples with some degree of methylation were found in regions A and B, which to some extent supports the hypothesis that methylation in these regions does not affect protein expression, and thus, does not influence MSI [13,14]. Moreover, they mentioned that the critical regions for protein expression loss are C (−248 to −178) and D (−109 to +5), as these contain a CCAAT sequence. Methylation in this area would inhibit the binding of the transcription factor CBF (Core Binding Factor), thereby inactivating gene expression [15,16].

Another study proposing regional analysis was conducted where five regions were analyzed: region A (−755 to −574), region B (−597 to −393), region C (−420 to −188), region D (−286 to −53), and region E (−73 to +86), reporting that 13 out of 210 patients with CRC showed total methylation of the *MLH1* CpG island, mainly observed in MSI-H samples (13/13), in contrast to partial methylation, which was found in only two MSI-H samples (13/2) (*p* < 0.0001) [14]. It is important to note that this is the first study that analyzes the five regions on *MLH1* in Mexico and the second worldwide while also examining their association with the microsatellite stability status.

Furthermore, it was suggested that *MLH1* methylation might serve as the second hit in hereditary cancer cases. Nevertheless, it could also act as the first hit in cases where methylation occurs without MSI, consistent with the progressive accumulation of genetic and epigenetic alterations characteristic of cancer [6]. In addition, three of the MSI-H samples did not exhibit methylation in any of the evaluated regions. This observation suggests that MSI in these cases may result from genetic alterations in other MMR system genes or alternative mechanisms of *MLH1* inactivation, as MLH1 deficiency is the most common cause of MSI. Considering the age of these patients (<50 years), the possibility of hereditary CRC should be considered. Previous studies have established an association between *MLH1* silencing through DNA methylation and the presence of MSI-H, which is a hallmark of the CpG Island Methylator Phenotype (CIMP) observed in CRC [41,42].

Finally, the limitation of the MS-PCR technique used in this study is that it focuses exclusively on CpG sites where primers hybridize. Therefore, using more advanced techniques, such as bisulfite sequencing, would provide more precise and quantitative data on methylation levels in these regions. Additionally, it would be important for future studies to evaluate how methylation of different regions on *MLH1* promoter relates to the modification of *MLH1* expression at the mRNA and protein level. Also, the MSI, as a genetic alteration, can result from defects in other genes within the DNA mismatch repair (MMR) system that were not evaluated in this study. Genes such as *PMS2*, *MSH6*, and *MSH2* may be implicated in tumors exhibiting MSI. This highlights the need for future studies aimed at identifying variants in these genes to better explain the presence of MSI in the analyzed cases.

## 5. Conclusions

Our study identified significant differences between *MLH1* methylation status and MSI. However, no differences were observed when analyzing specific methylation regions in relation to MSI. While previous research has investigated MSI in Mexican patients under 50 years of age, this study is among the first to describe the frequency of MSI independently of age, providing a broader understanding of its occurrence in this population.

## Figures and Tables

**Table 1:** Frequencies of clinical and pathological characteristics of patients with CRC (n = 138).

Characteristic		Frequencies (%)
Age *		57 years
Sex	Male	56.5
	Female	43.5
Location	Colon	32.3
	Rectum	67.7
Stage	I	7.9
	II	28.3
	III	45.7
	IV	18.1
Oncological background	Yes	55.4
	Not	44.6
Other *	Height (m)	1.63 (±0.12)
	Weight (kg)	68.2 (±20.5)
	BMI (kg/m^2^)	25.2 (±5.63)

* These results are shown on average. For quantitative data standard deviation is shown in parenthesis.

**Table 2:** Distribution of *MLH1* methylation across different categories of the MSA.

	UM n (%)	PM n (%)	M n (%)	Total n (%)	
MSS	100 (72.46)	26 (18.84)	2 (1.45)	128 (92.75)	
MSI-L	1 (0.72)	1 (0.72)	0 (0)	2 (1.45)	***p*** ** = 0.01**
MSI-H	3 (2.17)	2 (1.45)	3 (2.17)	8 (5.80)	
Total	104 (75.36)	29 (21.01)	5 (3.62)	138 (100)	

UM: Unmethylated, PM: Partially methylated, M: methylated, MSS: Microsatellite Stability, MSI-L: Microsatellite Instability Low, MSI-H: Microsatellite Instability High. *p* value was obtained with Fisher’s exact test. According to the Bonferroni method, the comparison between the M vs. MSS categories and M vs. MSI-H were responsible for the statistical significance, with *p*-values of 0.0233 and 0.0111, respectively.

## Data Availability

The original contributions presented in the study are included in the article, further inquiries can be directed to the corresponding author.

## Supplementary Materials

The following supporting information can be downloaded at: https://www.mdpi.com/article/10.3390/genes16020182/s1, Supplementary S1. *MLH1* gene amplification in regions A, B, C, D, and E., and primers for the amplification of five markers for microsatellite instability analysis. Supplementary S2. Results of MSI and methylation analysis by patient.
