# Supplementary material for: MLH1 Methylation Status and Microsatellite Instability in Patients with Colorectal Cancer
Source: Genes (Basel). 2025 Feb 2;16(2):182. doi: 10.3390/genes16020182 (PMC11854980; doi:10.3390/genes16020182)
Supplement: Supplementary file 1 [file genes-16-00182-s001.zip › Suplementary S1.pdf]

Suplementary 1

MLH1 gene amplification in regions A, B, C, D, and E

Region	M (Methylated)<br>U (Unmethylated)	Primers sequences	Fragment size (bp)
Region A<br>-677 to -539	M	F 5′-CGGTAGAGTTCGAGGTTTGTAC-3′<br>R 5′-CACGAATACTACGAACGATATATAACG-3′	134 bp
	U	F 5′ GTGGTAGAGTTTGAGGTTTGTATGA-3′<br>R5′-AAACACAAATACTACAAACAATATATAACA-3′	138 bp
Region B<br>-418 to -264	M	F 5′- GTCGGAAAATTAGAGTTTCGTC-3′<br>R 5′-GCAAAACGAAAAAAATACTTAACG-3′	151 bp
	U	F 5′- GGTTGGAAAATTAGAGTTTTGTTGA-3′<br>R5′-ACAAAACAAAAAAAATACTTAACACA-3′	154 bp
Region C<br>-162 to -62	M	F 5′-GATAGCGATTTTTAACGC-3′<br>R 5′-TCTATAAATTACTAAATCTCTTCG-3′	92 bp
	U	F 5′-AGAGTGGATAGTGATTTTTAATGT-3′<br>R 5′-ACTCTATAAATTACTAAATCTCTTCA-3′	100 bp
Region D<br>+88 to +260	M	F5′-GTTTTTTTGGCGTTAAAATGTC-3′<br>R 5′- CCTTAAATAAACCCGACTCGAC-3′	166 bp
	U	F5′ -TTGGTTTTTTTGGTGTTAAAATGTT-3′<br>R5′-AACCCTTAAATAAACCCAACTCAAC-3′	172 bp
Region E<br>+239 to +415	M	F 5′-GAGTCGGGTTTATTTAAGGGTTAC-3′<br>R 5′-GATAAAAAAACACACGATCTACGAA-3′	177 bp
	U	F 5′-AGTTGGGTTTATTTAAGGGTTATGA-3′<br>R 5′- AATAAAAAAACACACAATCTACAAA-3′	176 bp

Primers for the amplification of five markers for microsatellite instability analysis

Marker	Gen	Gene	Chromosome	Length and location of the repeat	Fluorophore	Primers sequence	Lenght (bp)
NR-27	*BIRC2*	Apoptosis inhibitor 1	11q22.2	27 (A)<br>5´UTR	HEX	F- 5´ AACCATGCTTGCAAACCACT 3´<br>R- 5´ CGATAATACTAGCAATGACC 3´	87
NR-21	*SLC7A 8*	Sodium solute carrier family 7-member 8	14q11.2	21 (T)<br>5´UTR	FAM	F- 5´ GAGTCGCTGGCACAGTTCTA 3´<br>R- 5´ CTGGTCACTCGCGTTTACAA 3´	109
NR-24	*ZNF2*	Zinc finger 2	2q11.1	24 (T)<br>3´UTR	NED	F- 5´ GCTGAATTTTACCTCCTGAC 3´<br>R- 5´ ATTGTGCCATTGCATTCCAA 3´	131
BAT-25	*KIT*	Proto-oncogene KIT	4q12	25 (T)<br>Intron 16	HEX	F- 5´ TACCAGGTGGCAAAGGGCA 3´<br>R- 5´ TCTGCATTTTAACTATGGCTC 3	153
BAT-26	*MSH2*	MutS Homolog 2	2p21-p16.3	26 (A)<br>Intron 5	FAM	F- 5´ CTGCGGTAATCAAGTTTTTAG 3´<br>R- 5 AACCATTCAACATTTTTAACCC 3´	183
